# The gray zone of the qualitative assessment of respiratory changes in inferior vena cava diameter in ICU patients

**DOI:** 10.1186/cc13693

**Published:** 2014-01-14

**Authors:** Antoine Duwat, Elie Zogheib, Pierre Grégoire Guinot, Franck Levy, Faouzi Trojette, Momar Diouf, Michel Slama, Hervé Dupont

**Affiliations:** Surgical Intensive Care Unit, Amiens University Hospital, Amiens, France; Medical Intensive Care Unit, Amiens University Hospital, Amiens, France; Department of Cardiology, Amiens University Hospital, Amiens, France; INSERM U-1088, Jules Verne University of Picardy, Amiens, France; Department of Innovation and Clinical Research, Amiens University Hospital, Amiens, France

## Abstract

**Introduction:**

Transthoracic echocardiography (TTE) is a useful tool for minimally invasive hemodynamic monitoring in the ICU. Dynamic indices (such as the inferior vena cava distensibility index (dIVC)) can be used to predict fluid responsiveness in mechanically ventilated patients. Although quantitative use of the dIVC has been validated, the routinely used qualitative (visual) approach had not been assessed before the present study.

**Methods:**

Qualitative and quantitative assessments of the dIVC were compared in a prospective, observational study. After operators with differing levels in critical care echocardiography had derived a qualitative dIVC, the last (expert) operator performed a standard, numeric measurement of the dIVC (referred to as the quantitative dIVC). Two groups of patients were separated into two groups: group (dIVC < 18%) and group (dIVC ≥ 18%).

**Results:**

In total, 114 patients were assessed for inclusion, and 97 (63 men and 34 women) were included. The mean sensitivity and specificity values for qualitative assessment of the dIVC by an intensivist were 80.7% and 93.7%, respectively. A qualitative evaluation detected all quantitative dIVCs >40%. Most of the errors concerned quantitative dIVCs of between 15% and 30%. In the dIVC <18% group, two qualitative evaluation errors were noted for quantitative dIVCs of between 0 and 10%. The average of positive predictive values and negative predictive values for qualitative assessment of the dIVC by residents, intensivists and cardiologists were 83%, 83%, and 90%; and 92%, 94%, and 90%, respectively. The Fleiss kappa for all operators was estimated to be 0.68, corresponding to substantial agreement.

**Conclusion:**

The qualitative dIVC is a rather easy and reliable assessment for extreme numeric values. It has a gray zone between 15% and 30%. The highest and lowest limitations of the gray area are rather tedious to define.

Despite reliability of the qualitative assessment when it comes to extreme to numerical values, the quantitative dIVC measurement must always be done within a hemodynamic assessment for intensive care patients. The qualitative approach can be easily integrated into a fast hemodynamic evaluation by using portable ultrasound scanner for out-of-hospital patients.

## Introduction

The management of patients with acute circulatory failure requires knowledge of the preload dependence state. Dynamic indices predict the response to volume expansion with greater accuracy than do static indices. This assessment is very important because inadequate fluid replacement or inappropriate infusion of catecholamines can lead to significant adverse effects [1]. Several of these dynamic indices (including those derived by echocardiography) have been validated in ICU patients [2–8]. Indeed, transthoracic echocardiography (TTE) is a valuable, minimally invasive, rapid hemodynamic monitoring tool for use in the ICU. TTE provides information not only on dynamic indices of preload dependence but also on overall contractility of the heart, filling pressures, and the impact of many diseases (septic shock, acute respiratory distress syndrome, cardiogenic shock, and so on). Echography for measuring hemodynamic parameters are easy to learn, and the corresponding indices have been assessed on several occasions [9–12]. Of these, the superior vena cava collapsibility index (cSVC) [5] and the inferior vena cava distensibility index (dIVC) have been widely validated [6, 7, 13–17].

Several studies have demonstrated the acceptability of qualitative (visual) measurement of the left ventricular ejection fraction when compared with quantitative reference measurements [18, 19]. Similarly, Vieillard-Baron *et al.*[20] validated the use of qualitative transesophageal echocardiography in the hemodynamic assessment of septic shock, which included the cSVC as an indicator of preload dependency. In clinical practice, many operators solely perform qualitative analyses of data from bedside echocardiography measurements, considered as valid as the quantitative measurement, and less time consuming. To the best of our knowledge, qualitative measurement of the dIVC in mechanically ventilated ICU patients has not been compared with quantitative measurement. Although this index is easily calculated on ultrasound with the use of the mean value from three measures [21–23], the threshold for discriminating between nonresponders and responders is small (12% or 18%, depending on the equation and the IVC analysis site used) [6, 7]. The quantitative assessment is completed by one individual operator who calculated the average of three measures. This can be considered time consuming.

The relevance and reproducibility of a qualitative approach for calculating the dIVC has not previously been established. We therefore sought to assess the accuracy of a qualitative approach of dIVC and compare it with that of the quantitative method. We also sought to evaluate the impact of the operator's level of experience on the accuracy of the qualitative approach.

## Materials and methods

### Patients

This was a prospective, observational study performed in a surgical ICU at a university hospital. The noninterventional study's objectives and data-collection procedures were approved by the Institutional Review Board (IRB) for human subjects at our hospital (N°20/2010 Commission d’Evaluation Ethique des Recherches Non Interventionnelles, Amiens University Hospital, Amiens, France). Informed consent was waived because the IRB considered the protocol to be part of usual care in clinical practice.

The main inclusion criteria were as follows: (a) patients undergoing TTE for hemodynamic assessment; (b) age older than 18 years; and (c) sedated and mechanically ventilated patients who were synchronized with the respirator (the Servo-i, Maquet, Germany). The main exclusion criteria were as follows: (a) patients with changes in hemodynamic status during the various measurement (changes in catecholamine doses, fluid expansion, and so on), and (b) poor visualization of the IVC by even the most experienced operator.

### Study protocol

The operators who performed a qualitative assessment of dIVC had various levels of experience and training in TTE: (a) four anesthesiology residents (*n* = 4), considered as operators with a basic level of experience (level 1) of critical care echocardiography (less than 30 TTEs), (b) two intensivists (*n* = 2) with an advanced level of experience (level 2) in critical care echocardiography, and (c) two cardiologists (*n* = 2) with high levels of expertise (level 3) in critical care echocardiography. The levels of echocardiography skills and knowledge were those described in the guidelines issued by the American College of Chest Physicians and the French Society of Intensive Care [9] and the international expert statement on training standards for critical care ultrasonography [10].

To avoid getting biased results by the different actors, the qualitative measure was completed by a resident, then an intensivist, and last by the cardiologist. The quantitative assessment was completed at the end of the data collection and solely by one individual who calculated the average of three measures.

### Qualitative evaluation

The IVC was visualized with a subcostal sonographic approach. Distensibility was assessed in M-mode (coupled to the 2D mode) just upstream of the junction of the hepatic veins, once the M-mode cursor was perpendicular to the IVC. The ultrasound system used was an EnVisor (Philips, Suresnes, France) with an S4-2 Hz probe. Operators performing echocardiographic examination of the IVC were blinded to the patient's fluid-responsiveness status. Each operator had to answer exactly three questions: Is the variation of dIVC greater than 18%, less than 18%, or not viewable?

### Quantitative evaluation

The dIVC was measured last by the most expert operator (that is, one of the two cardiologists). The image was obtained as described earlier, and the dIVC was calculated according to the following equation: (maximum diameter on inspiration _ minimum diameter on expiration)/minimum diameter on expiration. The speed of the trace was 12.5 mm/sec.

The quantitative reference cut-off value used for assessing preload dependence was 18%, in line with the study by Barbier *et al*. [7]. Patients with dIVC <18% were considered to be likely nonresponders in the event of fluid challenge. No fluid challenges were performed in our study. The quantitative dIVC analyzed here was the mean of three evaluations carried out successively by the same operator.

The respiratory pulse pressure variation (PP_variation_) was expressed as a percentage and calculated according to the following equation: PP_variation_ = ((systolic blood pressure (SBP)_max_ - diastolic blood pressure (DBP)_max_) - (SBP_min_ - DBP_min_))/((SBP_max_ - DBP_max_) + (SBP_min_ - DBP_min_))/2).

### Statistical analysis

Quantitative variables are expressed as mean ± standard deviation and compared with a Student *t* test. A *P* value <0.05 was considered statistically significant.

Qualitative variables are expressed as percentage and compared with χ^2^ or the Fisher test.

Clinical characteristics, ventilator settings, and hemodynamic parameters of patients with quantitative dIVC <18% were compared with patients with quantitative dIVC ≥18%. The sensitivity, specificity, positive predictive value, and negative predictive value of qualitative assessment of the dIVC were calculated. A kappa coefficient for interrater agreement was computed for each operator with respect to the reference value measured by an expert operator. We adopted the Landis and Koch conventions for describing the degree of agreement as a function of the value of kappa [24]. The statistical analysis was performed with the R.2.12.0 software (R Foundation).

## Results

### Patients

One hundred fourteen patients were screened. Because of poor visualization of the IVC by one or more of the operators, 17 (14.9%) of the 114 patients were not included in the study. Hence, our analysis covered 97 patients (63 men, 34 women; mean age, 67 ± 13 years; mean Simplified Acute Physiology Score II, 40 ± 13). The reason for ICU admission was postcardiac surgery care in 75 (77.3%) cases, septic shock in 18 (18.6%) cases, and multiple trauma in four (4.1%) cases. The patients were separated into two subgroups on this basis: (a) quantitative dIVC <18%; (b) quantitative dIVC ≥18%.

### Demographic and hemodynamic parameters

No differences were found between quantitative dIVC <18% and dIVC ≥18% groups in terms of demographic variables and severity scores (Table 1).

**Table 1:** **Clinical characteristics of the study population as a whole, group (dIVC <18%) and group (dIVC >18%)**

	All patients ( ***n*** = 97)	dIVC < 18% ( ***n*** = 71)	dIVC ≥ 18% ( ***n*** = 26)	***P*** value
Age (years)	67 ± 13	67 ± 13	68 ± 11	*0.57*
Weight (kg)	74 ± 15	75 ± 15	71 ± 14	*0.16*
Height (m)	1.69 ± 0.09	1.70 ± 0.08	1.67 ± 0.09	*0.1*
BMI (kg/m^2^)	25.6 ± 3.8	25.8 ± 3.8	25.2 ± 3.7	*0.45*
SAPS II	40 ± 13	40 ± 12	42 ± 15	*0.55*

*dIVC* inferior vena cava distensibility index, *BMI*, body mass index; *SAPS II,* Simplified Acute Physiology Score.

The main ventilator settings and hemodynamic parameters are summarized in Tables 2 and 3. As expected, responders had significant lower mean blood pressure (*P* = 0.04) and DBP (*P* = 0.03) values. The maximum and minimum IVC diameters were lower in responders (*P* < 0.01), and PPV was higher (*P* < 0.001).

**Table 2:** **Ventilator settings for the study population as a whole, group (dIVC <18%) and group (dIVC >18%)**

	All patients ( ***n*** = 97)	dIVC <18% ( ***n*** = 71)	dIVC ≥18% ( ***n*** = 26)	***P*** value
V_t_ (ml)	519 ± 45	518 ± 46	518 ± 46	*0.15*
RR (mn^-1^)	16 ± 3	16 ± 3	16 ± 3	*0.56*
PEEP (cmH_2_O)	4 ± 2	4 ± 2	4 ± 1	*0.3*
PPlat (cmH_2_O)	18 ± 5	18 ± 5	17 ±4	*0.56*
V_t_ (ml/kg)	8.3 ± 0.5	8.3 ± 0.4	8.3 ± 0.5	*0.1*

dIVC*,* inferior vena cava distensibility index; Vt , tidal volume; RR*,* respiratory rate; PEEP*,* positive end-expiratory pressure; PPlat*,* plateau pressure.

**Table 3:** **Hemodynamic parameters of the study population as a whole, group (dIVC <18%) and group (dIVC ≥18%)**

	All patients ( ***n*** = 97)	dIVC <18% ( ***n*** = 71)	dIVC ≥18% ( ***n*** = 26)	***P*** value
SBP (mm Hg)	120 ± 23	123 ± 23	114 ± 20	*0.1*
MBP (mm Hg)	82 ± 16	84 ± 17	77 ± 12	*0.04*
DBP (mmHg)	63 ± 15	65 ± 16	57 ± 12	*0.03*
HR (per min)	80 ± 16	81 ± 16	77 ± 18	*0.4*
Max IVC diameter (cm)	1.8 ± 0.4	1.9 ± 0.4	1.60 ± 0.4	*<0.01*
Min IVC diameter (cm)	1.6 ± 0.4	1.8 ± 0.4	1.2 ± 0.4	*<0.01*
PP_variation_ (%)	13 ± 7 (*n* = 82)	11 ± 5 (*n* = 61)	18 ± 9 (*n* = 21)	*<0.01*

dIVC*, inferior vena cava distensibility index*; SBP*, systolic blood pressure*; DBP*. diastolic blood pressure*; MAP*, mean arterial pressure*; HR*, heart rate*; PP_variation_ *, respiratory variation of pulse pressure.*

### Comparison of qualitative and quantitative analysis of IVC distensibility

The sensibility, specificity, positive, and negative predictive values were similar in different groups of operators and are presented in Table 4. The experienced and less experienced operators did not display significantly different kappa values.

**Table 4:** **Comparison of qualitative and quantitative inferior vena cava distensibility analysis by different operators**

	Se	Sp	PPV	NPV	Kappa
Residents (*n* = 4)	77%	94%	83%	92%	0.73
Intensivists (*n* = 2)	81%	94%	83%	94%	0.77
Cardiologists (*n* = 2)	69%	97%	90%	90%	0.72

Se*, sensitivity*; Sp*, specificity*; PPV*, positive predictive value*; NPV*, negative predictive value.*

The average sensitivity and specificity values for qualitative assessment of the dIVC by anesthesiologists (residents and intensivists) were 80.7% and 93.7%. The kappa coefficient for the entire group of operators was calculated to be 0.68, corresponding to substantial agreement. The kappa coefficients for each operator are presented in Table 4.

**Figure 1:**
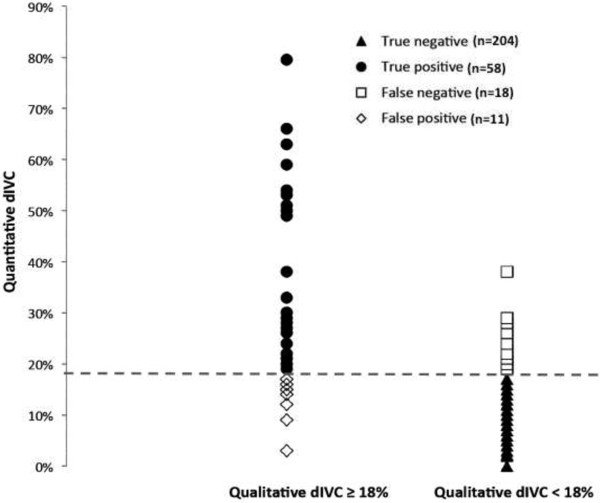
**Individual distribution of the quantitative inferior vena cava distensibility index for all operators, based on a qualitative assessment cut-off of 18%.**

**Figure 2:**
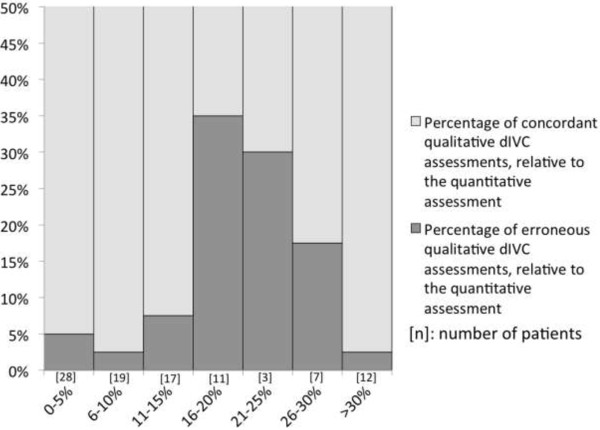
**Distribution of patients with an error in measurement of the qualitative inferior vena cava distensibility index, relative to the quantitative dIVC.** Values are expressed as the number of patients and the percentage of concordant assessments.

Qualitative dIVC assessment had a very low error rate for the 73 (75%) patients with quantitative values <15% and >30%. Unfortunately, the error rate was as high as 35% for quantitative values in the range 15% to 30%, which concerned 24 patients (25%) (Figures 1 and 2).

## Discussion

Our results showed that when compared with quantitative measurement and a threshold of 18%, qualitative assessment of the dIVC displayed good sensitivity and specificity and a substantial degree of interoperator agreement. This index was able to detect the majority of patients with a dIVC >30%.

Barbier *et al.*[7] reported that the increase in cardiac output during volume expansion is proportional to the measured value of dIVC. Hence, the qualitative assessment of dIVC in our study should have been able to detect (a) the majority of patients with a dIVC >30% and an increase in cardiac output of >15%, and (b) all patients with a dIVC > 40% and an increase in cardiac output of >20% during a fluid challenge test.

Similarly, this qualitative technique enables the detection of probable nonresponders to volume expansion with low error rates. Most of the errors in the qualitative dIVC evaluation occurred close to the threshold of 18%. The data in Figure 2 show that 90% of errors concerned patients with a dIVC of between 10% and 30%, and 65% concerned patients with a dIVC of between 15% and 25%.

As highlighted in the study by Vieillard-Baron *et al*. [20], norepinephrine may have limited efficacy in septic shock patients with hypovolemia or ventricular dysfunction. The study of Vieillard-Baron *et al.* found that experienced transesophageal echocardiography operators could assess these parameters qualitatively and that the provision of quantitative measures is potentially time-consuming. Our study gave similar results for the qualitative assessment of dIVC with TTE by operators with varying levels of experience. Thus, TTE can also yield a qualitative assessment of the dIVC, left ventricular ejection fraction, and right ventricular dysfunction.

The quality of the dIVC evaluation appeared to be good and did not depend greatly on the operator's level of echocardiographic experience. This finding is in agreement with the literature. Clinical work shows that it is possible to teach nonintensivists and noncardiologists how to perform basic transthoracic and transesophageal echocardiography [11, 12, 25] over a 12-month period by setting simple, standardized diagnostic goals. The principle of learning simple, standardized echocardiography skills goes beyond the field of intensive care. It has already been shown that a 4-hour course on the ultrasound analysis of inferior vena cava (with 20 clinical cases) can significantly improve the clinical diagnosis of vascular overload by residents in internal medicine [26]. Similarly, ultrasound analysis of the inferior vena cava makes it possible for nephrologists with little experience of ultrasound the better to assess blood-volume status between sessions for dialysis patients with chronic renal failure [27].

In the present study, the fact that the residents and intensivists did not differ significantly in terms of the recorded sensitivity and specificity values further suggests that learning is rapid. If used to evaluate hemodynamic status, serial quantitative measurement of the dIVC might help to improve the accuracy of qualitative analysis alone. Although it appears that the qualitative assessment of dIVC is easy to learn, further experience may increase the technique's reliability and reduce the error rate.

The cut-off used for assessing preload dependence with quantitative dIVC was 18% [7]: this is an arbitrary value that correctly evaluates the majority of patients. However, the application of a "gray zone" approach to PP_variation_ for prediction of fluid responsiveness in mechanically ventilated patients identifies a range of PP_variation_ values (from 9% to 13%) [28]. For dIVC evaluation, this gray zone is around 18%: volemic evaluation is difficult, and the benefit of fluid resuscitation is subject to debate. Just as with quantitative dIVC assessment, qualitative dIVC assessment is unreliable for patients with a volemic status in this gray zone; these patients should therefore receive an overall assessment (the clinical context, the passive leg-raising maneuver, respiratory pulse-pressure variation, and so on). With the exception of the gray zone (within which no single marker is sufficiently reliable), our study shows that the qualitative approach to dIVC assessment can be easily integrated into an overall hemodynamic assessment, when it comes to extreme to numeric values.

The quantitative dIVC requires less time with an evolutive ultrasonographic machine available in the intensive care units. The gain of accuracy of the quantitative dIVC measures has improved with the technique and can reduce its gray zone around 18%.

### Limitations

Our study has several limitations.

No fluid assessment was completed to find changes in qualitative dIVC or quantitative dIVC, nor in cardiac output. The qualitative dIVC was measured according to the hemodynamic status of patients when assessing different measures, nor was the study random, nor was it our main objective.

Our failure rate for visualization of the IVC was 14.9%. This rate is close to the literature values [29, 30]. However, our rates took account of all patients in whom at least one of the operators (with different levels of expertise) could not visualize the IVC (as recommended by the current guidelines). In addition, most of the admissions to our ICU are related to postcardiac surgery care; the presence of chest tubes can interfere with the operator's view and make it difficult or impossible to visualize the IVC.

Given that 14.9% of patients were not evaluable and 20.6% (20 patients) are in the gray area of dIVC, between 15% and 30%, about 40% of patients are not evaluable by reliable visual methods. Nevertheless, a limit exists for all assessments preload dependency with the inferior vena cava, because these limits also apply to quantitative dIVC if we consider the "principle of the gray area".

Although our qualitative assessment was performed by just eight operators with different levels of experience of TTE, the study involved a large enough number of patients to confirm the technique's utility under different load and volume-expansion conditions. However, the large number of measurements and the satisfactory correlations between qualitative and quantitative dIVC assessments by the various operators confirmed that it is possible to use the qualitative-technique assessment on a daily basis. Our least experienced operators (level 1) had performed an average of 30 TTE examinations. An operator must understand some basic technical details of TTE before he or she can perform a dIVC assessment, because it is not always easy to obtain a good view and identify the best measurement site. Validation of qualitative dIVC assessment by a larger number of operators would nevertheless be of value.

## Conclusion

The qualitative assessment of the dIVC has a gray zone between 15% and 30%. The sensitivity and specificity are good for values below 15% and above 30%, regardless of the operator's level of experience in ultrasound. Despite reliability of the qualitative assessment when it comes to extreme to numeric values, the quantitative dIVC measurement must always be done within a hemodynamic assessment for intensive care patients. The qualitative approach can be easily integrated into a fast hemodynamic evaluation by using a portable ultrasound scanner for out-of-hospital patients (evaluation of the dIVC, left or right ventricular dysfunction, and screening for tamponade or pleural effusion).

## Key messages

-  Qualitative assessment of the dIVC has a gray zone between 15% and 30%, which should encourage the use of quantitative measurement of the dIVC in intensive care patients.
-  The correlation between qualitative and quantitative dIVC was considered good regardless of the operator's level of experience in ultrasound.
-  Qualitative dIVC assessment can easily take place in out of hospital FAST hemodynamic assessment, with a portable ultrasound scanner.

## Abbreviations

BMI: Body mass index
cSVC: superior vena cava collapsibility index
DAP: diastolic arterial pressure
DBP: diastolic blood pressure
dIVC: inferior vena cava distensibility index
HR: heart rate
ICU: intensive care unit
IRB: Institutional Review Board
IVC: inferior vena cava
MAP: mean arterial pressure
NPV: negative predictive value
PEEP: positive end-expiratory pressure
PP: pulse pressure
PPlat: plateau pressure
PPV: positive predictive value
PP_variation_: variation respiratory pulse pressure
RR: respiratory rate
SAPS II: Simplified Acute Physiology Score
SBP: systolic blood pressure
Se: sensitivity
Sp: specificity
TTE: transthoracic echocardiography
V_t_: tidal volume.
